# Membrane-Elasticity Model of Coatless Vesicle Budding Induced by ESCRT Complexes

**DOI:** 10.1371/journal.pcbi.1002736

**Published:** 2012-10-18

**Authors:** Bartosz Różycki, Evzen Boura, James H. Hurley, Gerhard Hummer

**Affiliations:** 1 Laboratory of Chemical Physics, National Institute of Diabetes and Digestive and Kidney Diseases, National Institutes of Health, Bethesda, Maryland, United States of America; 2 Max Planck Institute of Colloids and Interfaces, Department of Theory and Bio-Systems, Potsdam, Germany; 3 Laboratory of Molecular Biology, National Institute of Diabetes and Digestive and Kidney Diseases, National Institutes of Health, Bethesda, Maryland, United States of America; Stanford University, United States of America

## Abstract

The formation of vesicles is essential for many biological processes, in particular for the trafficking of membrane proteins within cells. The Endosomal Sorting Complex Required for Transport (ESCRT) directs membrane budding away from the cytosol. Unlike other vesicle formation pathways, the ESCRT-mediated budding occurs without a protein coat. Here, we propose a minimal model of ESCRT-induced vesicle budding. Our model is based on recent experimental observations from direct fluorescence microscopy imaging that show ESCRT proteins colocalized only in the neck region of membrane buds. The model, cast in the framework of membrane elasticity theory, reproduces the experimentally observed vesicle morphologies with physically meaningful parameters. In this parameter range, the minimum energy configurations of the membrane are coatless buds with ESCRTs localized in the bud neck, consistent with experiment. The minimum energy configurations agree with those seen in the fluorescence images, with respect to both bud shapes and ESCRT protein localization. On the basis of our model, we identify distinct mechanistic pathways for the ESCRT-mediated budding process. The bud size is determined by membrane material parameters, explaining the narrow yet different bud size distributions *in vitro* and *in vivo*. Our membrane elasticity model thus sheds light on the energetics and possible mechanisms of ESCRT-induced membrane budding.

## Author Summary

Lipid membranes enclose the cytosol of biological cells and compartmentalize their interior. Vesicles are used to transport membrane proteins between cellular compartments. The ESCRT protein machinery induces the creation of such vesicles away from the cytosol. The resulting vesicles are uncoated by protein. Upon vesicle scission and release into the endosome, the ESCRT proteins are recycled into the cytosol. We develop a membrane-elasticity model that captures this budding process. The model reproduces the vesicle morphologies observed in fluorescence microscopy images, and identifies the energetic driving force of vesiculation. We also characterize possible mechanisms of ESCRT-induced membrane budding. The size of the resulting vesicles is determined by membrane material parameters, explaining the narrow yet different bud size distributions *in vitro* and *in vivo*. Our membrane elasticity model thus provides insight into the energetics and mechanisms of uncoated vesicle formation.

## Introduction

**Figure 1:**
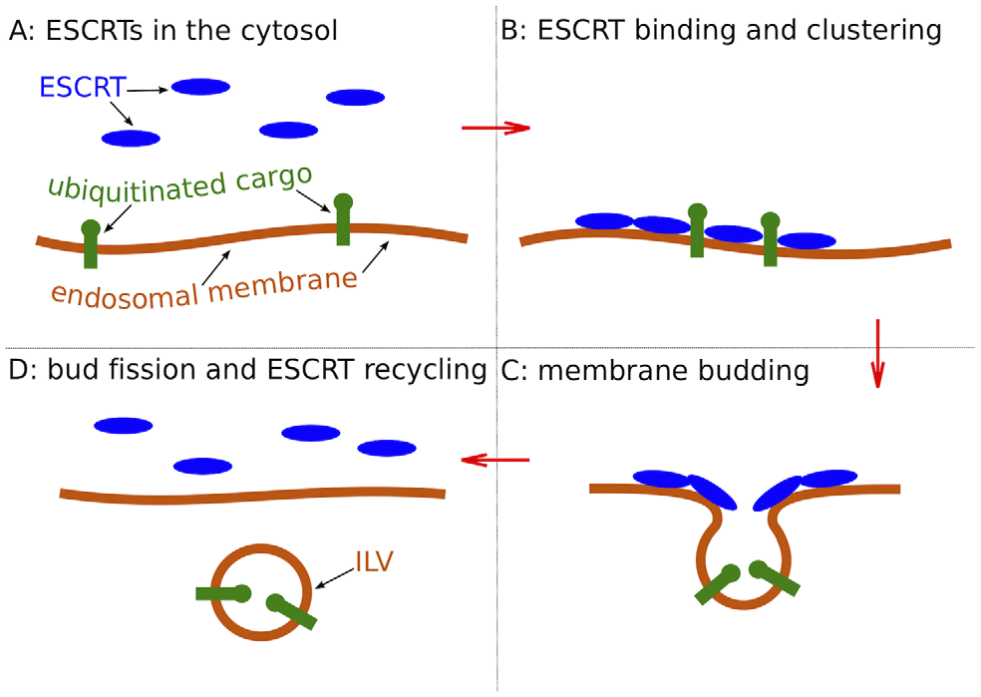
ESCRT protein assemblies (blue) facilitate the sorting of ubiquitinated membrane proteins (green) and formation of intralumenal vesicles (ILV). The ESCRT proteins assemble on the membrane side opposite the vesicle bud (C) and do not enter the ILV lumen for possible recycling (D). Energy input from ATP hydrolysis accelerates disassembly of the ESCRT machinery (D), but is not required in steps (A–C).

Lipid membranes enclose the cytosol of biological cells and compartmentalize their interior. The structure and contents of cellular membranes are actively controlled to sustain the vital functions of the cell. Transport vesicles are used to traffic membrane-bound proteins between cellular compartments. The best-characterized pathways of vesicle formation include those facilitated by BAR domain proteins [1] and by the coat protein clathrin with its adaptors [2]. Coat proteins impose their curved shape onto the membrane, thereby promoting vesicle curvature in an intuitively straightforward and computationally well-characterized process [3]–[5]. In the degradative transport of membrane proteins from endosomes to lysosomes (Fig. 1), small patches of the endosomal membrane bud into the interior (lumen) of the endosome and detach, forming intralumenal vesicles (ILVs) [6], [7]. This pathway is catalyzed by the cytosolic Endosomal Sorting Complex Required for Transport (ESCRT) [8]–[10]. The ESCRT proteins are not internalized in ILVs, but rather recycled continuously in the cytosol [11], [12]. To avoid the consumption of ESCRT proteins within the ILVs, these vesicles contain no protein coat to template their shape. Rather, these vesicles are initially formed as buds whose necks are stabilized by an assembly of ESCRT-I and -II [13]. As the assembly matures with the incorporation of ESCRT-III, the bud neck is cleaved from the cytosolic side, leaving ESCRTs in the cytosol and the detached spherical ILVs in the lumen of the endosome. Because this process involves no protein coat, the shape and energy of the mature buds must be governed primarily by membrane mechanical properties.

Until recently, the membrane remodeling functions of ESCRT were attributed only to ESCRT-III proteins, which assemble on liposomes *in vitro* [14], induce the ILV formation in giant unilamellar vesicles (GUVs) [12] and cause the plasma membrane to bud and tubulate when overexpressed in cells [15]. Based on these experimental observations, membrane deformations induced by ESCRT-III have been modeled [12] and studied theoretically [16]. An unresolved problem of these models was their inability to explain how the ESCRT-III proteins can be recycled from self-induced buds or tubes.

Recent *in vitro* experiments showed, however, that ESCRT-I and -II together are responsible for vesicle budding [13]. In these experiments, vesicle budding could be induced at physiological concentrations (15 nM) of ESCRT-I and -II. Fluorescence microscopy showed ESCRT-I and -II to be colocalized in the neck region of the buds (Fig. 1C), where they were shown to recruit ESCRT-III. The latter proteins then induced membrane scission [12]. Importantly, the ESCRT proteins were not found in the bud lumen. In this way, the ESCRT machinery that facilitates membrane budding and scission is not consumed in the process of ILV formation (Fig. 1D). Moreover, with ESCRT binding restricted to the neck region, the principal part of vesicle buds *in vitro* is thus bare lipid membrane.

Fluorescence microscopy [13] also showed that membrane-bound ESCRT proteins form microdomains on vesicle membranes (Fig. 1B and 1C). The lipid composition of these domains likely differs from that in the ESCRT-free membrane portions because ESCRTs bind specifically to certain charged lipids (PI3P) and could employ raft-favoring lipids and cholesterol to facilitate membrane budding [17], [18].

These two experimental observations of (i) the formation of ESCRT microdomains on the membrane and (ii) the formation of coatless buds (with ESCRT proteins localized in the bud neck) jointly form the basis for a phenomenological model of ESCRT-induced budding. Observation (i) provides us with the starting point for the budding process and motivates a mechanism that can provide the large energy necessary for membrane deformation. Observation (ii) determines the end point of the budding pathway. In our model, we assume that the ESCRT-I-II supercomplexes have an enhanced affinity for binding to saddle-shaped membrane regions, and that a line tension acts on the outer boundary of an ESCRT-sequestered membrane domain. We cast our model in the framework of membrane elasticity theory. A bending elastic model of lipid bilayers was previously used to study the energetics of a possible mechanism of ESCRT-III induced fission of nascent vesicles [19]. Here we employ a similar approach to study the ESCRT-I-II induced formation of vesicle buds.

The analytical solutions of our model allow us to map out different membrane morphologies over a range of possible physical parameters. We identify a regime of membrane bending parameters and line tensions for which the minimum energy configurations are coatless membrane buds with ESCRTs localized in the bud neck. These minimum energy configurations closely resemble the fluorescence images observed in experiment [13]. Within our model, we also identify energetically and kinetically feasible budding pathways, and propose a three-stage mechanism of ESCRT-driven budding: (i) membrane-bound ESCRT-I-II complexes form clusters, or domains, and induce a line tension on the domain boundaries through local segregation of lipids; (ii) as the domain boundary energy exceeds a threshold level, the membrane patch sequestered by the ESCRT assemblies buckles and forms a bud; (iii) the ESCRT-I-II complexes scaffold the bud neck and thus stabilize a neck diameter optimized for ESCRT-III protein binding and bud scission. To validate the model, we compare its predictions for bud shapes, sizes, and formation kinetics to experiment [13], [20]. We also relate our model to recent experiments probing ESCRT-induced lipid segregation in membranes [21].

## Model

In the framework of membrane elasticity theory, the energy of membrane deformations consists of the mean curvature term
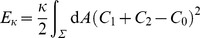
(1)and the Gaussian curvature term
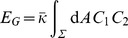
(2)where 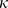 and 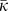 are the mean and Gaussian bending rigidity moduli, respectively; 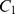 and 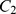 are the principal local curvatures; 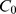 is the spontaneous curvature; and the integrals are performed over the membrane surface 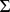 [22]. For uniform membranes with fixed topology, the energy term 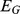 does not depend on membrane shape. Consistent with *in vitro* experiments [13], we will consider symmetric lipid bilayers with no spontaneous curvature, 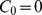.

Eq. (1) implies that the energetic cost of forming a vesicle bud [23]–[27] is approximately 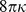, which exceeds 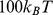 for typical membrane bending rigidities 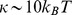. This large energetic cost effectively eliminates random thermal fluctuations as a main factor. Instead, the energetic cost of the nascent vesicle bud has to be offset by the molecular machinery that drives the budding process. In ESCRT-induced budding, the source of this offset energy is provided by the binding of the ESCRT proteins to the membrane. But unlike BAR domains or clathrin proteins, the ESCRT machinery does not seem to use the scaffold mechanism as the primary mode of action. Indeed, the scaffold mechanism cannot easily account for the significantly different bud radii *in vitro* (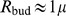
m [13]) and *in vivo* (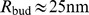 [20]), and appears to be contradicted by the experimental observation that the ESCRT proteins colocalize in the neck of buds.

How does the ESCRT machinery deform a flat membrane patch into a bud [28]? There are two relevant observations that help answer this question. Firstly, there is increasing evidence that the insertion of amphipathic, cationic peptide segments into the lipid bilayer can induce negative Gaussian curvature [29]–[31], which is a characteristic of the bud necks where the ESCRT proteins localize. The Vps37 subunit of ESCRT-I has such a cationic helix at its N-terminus [32], as does the Vps22 subunit of ESCRT-II [33]. It is therefore possible that the ESCRT-I-II proteins generate negative Gaussian curvature by inserting amphipathic and cationic segments into the membrane. In the lowest order of approximation, the resulting curvature-dependent binding energy,
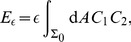
(3)is proportional to the membrane Gaussian curvature 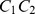 integrated over an ESCRT-rich membrane patch 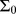, where 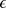 is a coupling constant. Remarkably, we also arrive at an energy term that is formally equivalent to Eq. (3) on an entirely different route. If we assume that by binding to the membrane, the ESCRT-I-II proteins locally perturb its Gaussian bending modulus according to Eq. (2), then the parameter 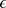 can be understood as the Gaussian bending modulus contrast 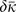. The energy term in Eq. (3) thus accounts for both the preferential binding to saddle-shaped features of the membrane and for changes in the Gaussian bending modulus in response to binding, with different interpretations of the constant 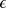.

The second relevant observation is that late endosomes contain raft-like domains that are rich in cholesterol and sphingomyelin [34]–[36]. The *in vitro* experiments [13] were performed on vesicles containing cholesterol, raft-favoring lipids and PI3P, which is consistent with the possibility that ESCRTs could use lipid rafts or domains to promote membrane budding [17], [18]. The ESCRT proteins could bind to pre-existing rafts or induce lipid domain formation. In fact, proteins with multiple lipid binding sites can induce lipid segregation in membranes close to the demixing point [37]–[40]. Lipid segregation has been suggested as a factor driving scission in endocytosis [41]. The enhancement of lipid segregation upon protein binding is primarily an entropic effect. Lipid segregation has to overcome an entropic penalty to move away from a perfectly mixed state. This penalty is reduced by concentrating certain lipids through preferential binding because the entropic cost of rearranging the resulting clusters of protein-bound lipids is smaller than that of rearranging individual lipids.

We consider a single lipid-segregated domain 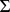. A line tension 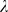 acts on the boundary 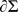 of this domain, where the corresponding energy is proportional to the length of the boundary line
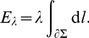
(4)To quantify the preferential binding of ESCRTs to lipid rafts or domains, we introduce an additional energy term in the spirit of mean field theory
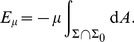
(5)Here, 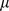 is an effective ESCRT binding free energy per unit area, and 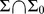 denotes the intersection of the lipid-segregated domain 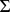 and the ESCRT-rich domain 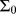 on the membrane, where we assume that the latter is a subset of the former, 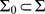. In the following, we assume the shapes of 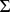 and 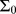 to be circular and ring-shaped, respectively.

As we show below, a minimal model of ESCRT proteins inducing or enhancing lipid segregation can explain central experimental results, in particular the narrow yet different bud-size distributions *in vivo* and *in vitro*. Moreover, the line tension on the boundaries of the lipid-segregated membrane domains [42]–[44] can also provide the large energetic driving force for vesicle budding [45]–[48].

Our model is defined by the energy functional
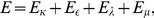
(6)which combines the energy terms in Eqs. (1), (3), (4), and (5). The four corresponding parameters 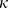, 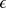, 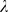, and 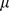 depend on membrane lipid composition, on external conditions such as temperature, and on ESCRT molecular properties, which are not entirely understood yet. Therefore, the model parameters can take different values depending on the molecular details of the ESCRT-membrane system. We thus use the spherical cap approximation [45], [49] to derive analytical solutions that allow us to map out the entire parameter space of the model. We then minimize the energy functional Eq. (6) with respect to the membrane shape and the ESCRT cluster location, and investigate the membrane states, or morphologies, that are stable in different parameter regimes. Interestingly, we find a range of physically meaningful parameters in which the minimum energy states closely resemble the fluorescence images in Ref. [13]. We also investigate possible budding pathways that lead without activation barriers from a flat membrane state to the coatless membrane bud with ESCRTs localized in the neck.

## Results

In the following, we first develop an approximate analytic treatment that accurately captures the energetics. We then report the results of numerical calculations that allow us to test the analytical solutions and to provide detailed information about the bud shapes. Finally, we analyze possible budding pathways.

### Energetics and morphology diagrams

**Figure 2:**
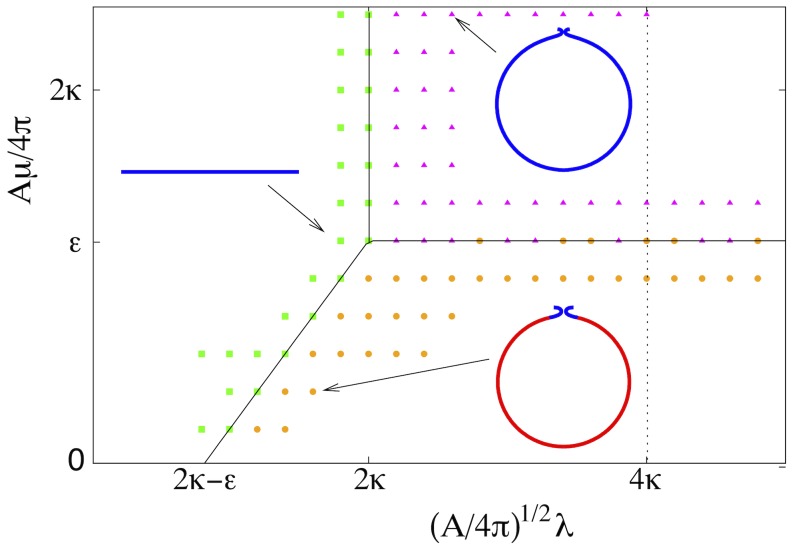
Membrane morphology diagram from the analytical solution obtained for the spherical cap approximation (lines) and from numerical energy minimizations (symbols). Results are shown for a Gaussian bending modulus fixed at 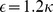. The green squares represent the flat membrane state. The magenta triangles correspond to ESCRT-coated membrane buds. The orange circles represent bare lipid buds with ESCRTs localized in the bud neck. The black solid lines are the coexistence lines, and the black dotted line represents the spinodal at which the energetic barrier to bud formation vanishes. Exemplary membrane shapes are shown for indicated points. The red and blue lines represent lipid domains and ESCRT-rich domains, respectively.

To make our model analytically tractable, we applied the spherical cap approximation, which is known to capture the energetics of unassisted membrane budding [45], [49]. In the spherical cap approximation, the energy functional Eq. (6) becomes a simple function whose minima can be found analytically (see Methods). The phase diagram that results from the energy minimization for 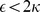 displays three regimes which are shown in Fig. 2. For small line tensions 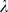 (left side of Fig. 2), the membrane resists bending and remains flat. The lipid-segregated domain 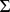 and the ESCRT-rich domain 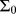 colocalize entirely. For large line tensions 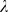 and large binding energies 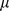 (top right corner of Fig. 2), the membrane buckles and forms an ESCRT-covered bud. Again 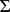 and 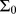 colocalize. For large 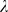 and small 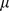 (bottom right corner of Fig. 2), the minimum energy configurations are coatless buds with ESCRTs localized in the bud neck.

### Membrane shape

To test the spherical cap approximation and to calculate bud shapes, we minimized the energy functional Eq. (6) numerically with respect to the membrane shape and the location of the ESCRT cluster (see Methods). To distinguish different morphologies of the minimum energy configurations, we introduced the area fraction 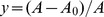, where 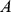 and 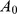 denote the areas of the lipid segregated domain 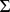 and the ESCRT-rich domain 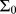, respectively. We grouped all the minimum energy configurations found numerically into three distinct categories: (i) flat membrane domains with zero bending energy and area fraction 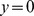, (ii) ESCRT-coated buds with bending energy 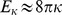 and area fraction 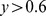, and (iii) coatless buds with 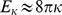, small area fraction 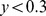, and the ESCRT cluster located primarily in the bud neck region. The resulting morphology diagram agrees very well with the diagram based on the spherical cap approximation (Fig. 2).

**Figure 3:**
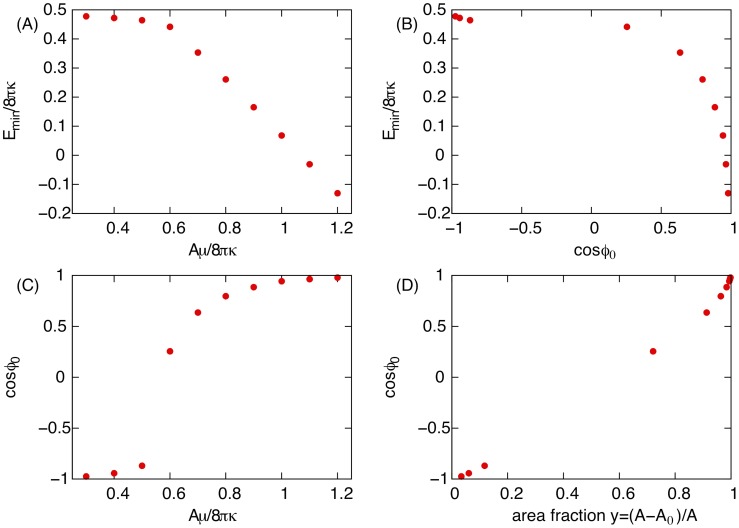
Relation between energetic and geometric properties, obtained by numerical minimization of the energy functional Eq. (6) for fixed parameters 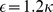 and 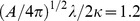. (A) Minimum energy 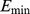 rescaled by the membrane bending rigidity 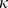 as a function of the ESCRT binding energy 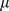 rescaled by the area 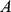 of the lipid-segregated domain. (B) Minimum energy 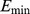 as a function of the cosine of the membrane tangent angle 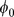 along the lines of longitude at the boundary of the ESCRT-coated domain. (C) 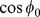 as a function of the binding energy 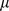 rescaled by the area 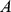 of the lipid-segregated domain 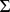. (D) 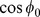 as a function of the area fraction 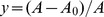, where 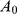 is the area of the ESCRT-rich domain 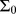.

The spherical cap model predicts discontinuous transitions between the different morphological phases, with a kink in the minimum energy at the transition lines. In the complete model, however, the kink is rounded at the transition line 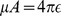 due to the finite-size of the bud neck (Fig. 3A).

To additionally quantify the bud morphologies, we determined the tangent angle 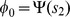 (see Methods) along the longitude at the boundary of the ESCRT-coated domain. The angle 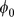 as well as the area fraction 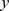 exhibit an abrupt change at the transition line 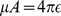, as shown in Fig. 3, which further justifies the distinction between ESCRT-coated buds and uncoated buds.

It is instructive to have a closer look at the membrane buds shown in the bottom right corner of Fig. 2. The principal part of these buds is coatless membrane, and the ESCRT cluster is localized only in the bud neck. These coatless buds assume the shape of a sphere. The geometry of the bud neck follows that of a catenoid, a minimal surface with no mean curvature, 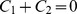, and thus zero bending energy [24]–[27]. As a result, the bending energy of the buds approaches the bending energy of a spherical vesicle, 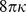. We note that our calculated bud shapes, as shown in the top right corner of Fig. 2, resemble the tomographic images of early endosomes [20] and the fluorescence images of GUV buds observed *in vitro* [13].

### Budding mechanisms

Within the framework of the spherical cap approximation, we also analyzed possible budding pathways. We focused in particular on activation-less pathways along which the buds grow without having to cross significant energy barriers (see Methods). This analysis revealed a three-stage mechanism of ESCRT-mediated membrane budding. In stage (i) the ESCRT-rich domain 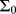 and the lipid-segregated domain 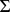 colocalize entirely on the flat membrane if 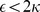. As the domain radius increases beyond 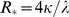, the energy of the flat domain becomes larger than the energy of an ESCRT-covered bud. As the domain radius increases to 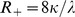, the barrier between these membrane states vanishes entirely and the system enters into stage (ii) in which the flat membrane domain buckles and forms an ESCRT-covered bud. The resulting bud has a radius
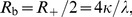
(7)which, interestingly, is determined by membrane material parameters. In stage (iii) the ESCRTs coalesce into the bud neck with no energy barriers if 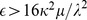. The third stage is driven by the energy 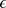 of the ESCRT preferential binding to membrane regions with negative Gaussian curvature. Importantly, in stage (iii), the bud is cleared of ESCRTs for possible recycling.

If 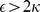, the mechanism of ESCRT-mediated membrane budding is different. The flat membrane with ESCRTs bound is then unstable and the system evolves spontaneously to a transient state, in which ESCRT-free buds with radius 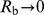 are formed. The mini-buds, induced by a strong binding preference 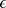 to saddle-shaped membrane structures, are next squeezed bigger by line tension 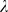 if the radius of the ESCRT-coated membrane patch is smaller than 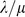. The latter condition puts an upper bound on the ESCRT-free bud radius 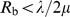. Interestingly, on this budding pathway the ESCRTs never coat the bud. We note, however, that this pathway is somewhat problematic since the linear approximation in Eq. (3) may not be applicable for large parameters 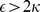. In addition, effects of finite membrane thickness cannot be ignored in this regime.

## Discussion

The detailed physical mechanisms used by the ESCRT machinery to control membrane budding are currently unknown. This lack of knowledge has motivated us to consider a simple, phenomenological model for ESCRT-induced membrane budding. Our model is based on well-established physical principles to ensure robustness. Following earlier studies of membrane tubulation [16] and fission [19] driven by ESCRT-III proteins, we have cast our model in the framework of membrane elasticity theory. The key assumption in our model is that ESCRTs induce or enhance the formation of raft-like domains in lipid membranes. Indeed, recent total internal reflection fluorescence (TIRF) images show that the ESCRT-II proteins induce lipid phase separation in model membranes with endosome-like composition [21]. These direct experimental observations thus support a central element of our theoretical model. However, to test and further quantitate our model will require additional studies of ESCRT-membrane interactions. In particular, line tension measurements would provide critical input in building a fully quantitative description of the budding process. In addition, studies of the different components of the ESCRT system, including modifications such as Vps20 myristoylation, should provide further guidance at which stage and to what extent lipid phase separation is induced. The TIRF experiments [21] indicate interesting variations, with the yeast ESCRT-II protein complex forming somewhat smaller and more numerous clusters than the human ESCRT-II protein complex under the same conditions. Ultimately, it will be important to determine if membrane curvature is induced in a way consistent with our theoretical analysis. Such measurements would provide critical tests to what extent our model captures ESCRT-induced budding, and would help in its quantitation and refinement.

The energetic analysis of our model suggests a three-stage mechanism of vesicle budding induced by the ESCRT proteins. In the first stage, the ESCRT-I-II binding induces lipid phase segregation, consistent with the TIRF experiments [21]. We speculate that in this stage, ESCRT-I-II complexes loosely assemble on the membrane to form clusters or domains. The ESCRT-membrane interactions enhance lipid segregation in the membrane and induce a line tension 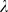 on the domain boundaries. Once the lipid-segregated membrane domain exceeds a critical size, as determined by membrane material parameters according to our model, the process enters the second stage. Beyond a size threshold set by the ratio 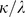, the energy barrier to membrane shape deformation disappears, and the membrane patch sequestered by ESCRTs buckles and forms a bud. In the third stage, according to our energy function, the ESCRT-I-II complexes concentrate in the neck of the newly formed bud. This final step is driven the ESCRT binding energy 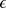 to the membrane portions with negative Gaussian curvature. While our model lacks molecular detail of this curvature-dependent binding, recent experiments [29]–[31] suggest possible mechanisms. In particular, it could be caused by the insertion of amphipathic and cationic segments of the membrane-bound ESCRT proteins into the membrane, or by the formation of an assembly whose shape is complementary to that of the bud neck [50]. The formation of neck-coating assemblies by the ESCRT-I-II complexes not only would help recruit ESCRT-III proteins for scission, but could also clear the ESCRT-I-II proteins from the bud lumen and set them up for recycling.

The third stage of the budding process described above is dynamically feasible within the overall time scale of the process. The diffusion coefficient of membrane proteins is of order 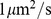 [51]. Provided a gradient to the bud neck region, the process of clearing *in vitro* buds with radius 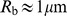 [13] would be completed within 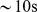. The clearing process would be two orders of magnitude faster for the *in vivo* buds with radius 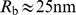 [20].

One immediate consequence of the three-stage mechanism is that the complete buds have a radius 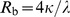 that is determined by membrane material parameters, namely membrane rigidity 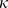 and line tension 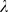. Therefore, the bud radius 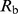 attains a particular value for a vesicle at given conditions, which explains the narrow distribution of bud sizes observed both *in vitro* [20] and *in vivo* [13]. We note, however, that both the line tension 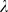 and the bending rigidity 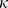 may be affected by details of the ESCRT-membrane interactions, which could explain small ILV size variations in response to mutations [52].

Despite the 40-times smaller radius 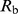, the *in vivo* buds have the same bending energies and shapes as the buds observed *in vitro*. This conclusion is a direct consequence of the scale invariance of the membrane bending energy Eq. (1). However, the line tensions necessary to induce vesicle budding, 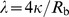, are different. For *in vivo* buds with radius 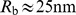 and bending rigidity 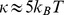 [53], we estimate 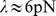; for *in vitro* buds with 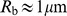, we get 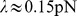 assuming the same membrane rigidity 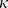. Line tensions in this range have been measured in GUVs [42], [47].

We note that the bending rigidity of early endosomes [53] is an order of magnitude lower than the bending rigidity of lipid membranes in the liquid-ordered phase [47], [54]. This difference in mechanical properties may be caused by variations in the composition and by the presence and activity of proteins in the membrane of endosomes. The membrane-sculpting protein Sar1 of the COPII complex, for example, has been shown to lower the membrane rigidity below 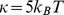 [55]. The rigidity of fluid membranes has also been shown to decrease gradually with increasing concentration of the HIV-1 fusion peptide in the bilayer [56].

The three-stage budding process discussed above occurs if the Gaussian bending modulus contrast 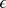 is limited to a window 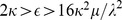. This relation puts an upper bound on the binding energy 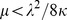. For typical bending rigidities 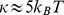 and line tensions 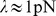, the required energy 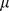 is of order 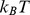 per 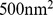 or smaller.

If the affinity of ESCRTs to regions with negative Gaussian curvature is high, 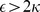, our model predicts a different budding pathway. In this case, flat ESCRT-coated membrane domains are unstable, and small ESCRT-free buds form spontaneously. The mini-buds induced by a strong binding preference 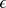 to saddle-like membrane shapes can next be squeezed bigger by line tension 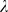. The latter transition occurs if the radius of the ESCRT-coated domain does not exceed 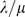. The resulting ESCRT-free buds have a radius 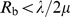. For 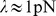 and 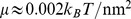 as above, we get the bud radius 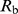 smaller than approximately 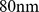, which is consistent with the size of late endosome buds, 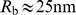 [20].

In contrast to the three-stage mechanism, in the pathway starting with mini-buds the ESCRTs never enter the bud lumen. It is thus tempting to picture this alternative budding route as follows: ESCRT-0 and -I bind to the membrane with 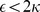 and the membrane remains flat. When ESCRT-II proteins bind to ESCRT-I and to the membrane, 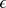 becomes larger than 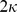 and the ESCRT-free membrane buds are formed spontaneously. The condition for forming a finite-size, ESCRT-free bud is that the radius of the ESCRT patch at the ESCRT-II arrival can not exceed 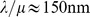. We notice, however, that this pathway might be an artifact of our model since the linear approximation in Eq. (3) could be inapplicable for large 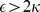. Nevertheless, we cannot entirely exclude this alternative pathway.

Overall, we favor the three-stage mechanism as a possible route for ESCRT-induced budding. It concisely explains the uniform bud sizes seen in experiment, and makes quantitative and testable predictions for their dependence on physical parameters. In particular, according to Eq. (7) the vesicle bud radius 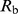 should increase linearly as a function of the ratio 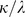. Since the line tension 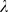 depends sensitively on temperature [42], it should be possible to test this theoretical prediction in future experiments on GUVs.

A key element of the model is the Gaussian bending energy 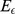. Because of experimental difficulties, our understanding of Gaussian curvature effects is rather limited. However, factors affecting the Gaussian bending modulus [57], [58] or enhancing the binding to membranes with negative Gaussian curvature [29]–[31] have been established. As detailed structural models of the full-length ESCRT protein supercomplexes emerge [50], [59], it will be important to identify the interactions responsible for the preferential association of the ESCRT proteins to saddle-shaped bud-neck membrane regions. It may also be possible to study binding of ESCRT proteins or protein fragments to model systems with negatively curved membranes [29]–[31]. In this way, one could gain a microscopic quantification of the parameter 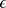, putting it on similar footing as the other membrane-bending terms.

## Methods

We consider only axisymmetric vesicle buds as the lowest energy shape by symmetry. Their surface is described in terms of two parameters, the angle 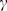 of rotation around the axis of symmetry, and the arc length 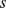 along longitudes. The vesicle bud surface then has Cartesian coordinates 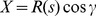, 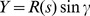 and 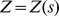, where 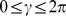 and 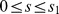 and 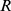 is the distance from the 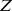-axis, which is the axis of symmetry of the bud. One can introduce a tangent angle 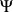 such that 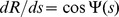 and 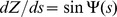. In this parametrization, the principal local curvatures are 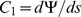 and 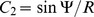 [60], [61]. Therefore, in this parametrization, Eq. (1) reads
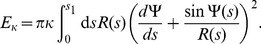
(8)The lower limit of integration, 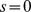, corresponds to the “south pole” of the vesicle bud at which 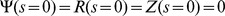. With these boundary conditions we have 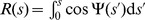 and 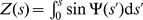. The upper limit of integration in Eq. (8), 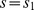, corresponds to the bud rim, where 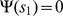 and 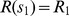 to connect the bud smoothly to a flat membrane at a distance 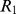 from the axis.

The boundaries of the ESCRT-occupied membrane patch are located at 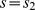 and 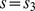 with 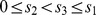. Eq. (3) takes the following form in the arc-length parametrization,
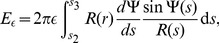
(9)which simplifies to
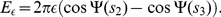
(10)In this parameterization, the energy terms in Eqs. (4) and (5) become
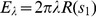
(11)and
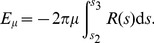
(12)The total energy is 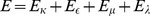.

### Numerical calculations

It is convenient to use dimensionless variables in numerical calculations. Here we introduce
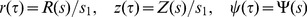
(13)and 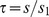 with 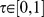. With these dimensionless variables, the boundaries of the ESCRT-occupied membrane patch are described by parameters 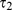 and 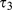, where 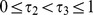. The energy functional
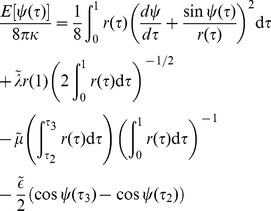
(14)depends then on the bud shape, which is described by
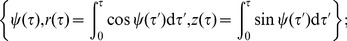
(15)on the domain location as given by 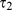 and 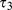; and on three dimensionless parameters
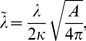
(16)

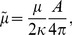
(17)and
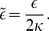
(18)In deriving the equations above we used a relation between the contour length 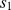 and the membrane area 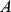, namely
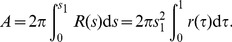
(19)The membrane energy (14) is minimized numerically with respect to the bud shape 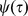 and the domain boundaries 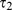 and 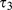 using a simulated annealing method. To this end, the function 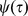 is assumed to be smooth at 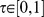 and approximated by a Fourier series
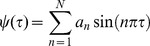
(20)that fulfills the boundary conditions 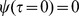 and 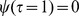. Here, 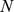 is the number of Fourier amplitudes 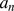. In simulated annealing Monte Carlo moves, the amplitudes 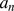 and the boundary positions 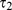 and 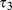 are varied. We performed the numerical calculations with up to 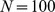 Fourier modes as in [62], [63]. For any set of parameters 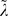, 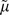, and 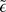, numerical calculations were repeated twice with different initial configurations to ensure convergence to the global minimum.

### Spherical cap approximation

To make our model analytically tractable, we approximate the nascent membrane bud by a spherical cap of radius 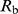 with a budding angle 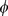. A convenient budding coordinate is 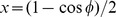. We also define the area ratio 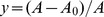 where 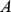 and 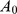 denote the areas of the lipid-segregated domain 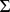 and the ESCRT-rich domain 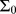, respectively. The domain of the two dimensionless parameters 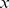 and 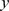 is the unit square. In the following, we write the energy of the bud in terms of 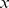 and 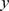, considering two cases:

**Case I:** The whole lipid-segregated domain 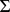 forms a spherical cap and thus the membrane surface parametrization is given by 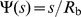 for 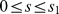 with 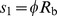. Then 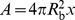 and the energy functional Eq. (6) becomes a two-variable function
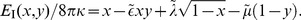
(21)


**Case II:** Only the ESCRT-free membrane patch forms a bud and therefore the membrane surface parametrization is given by 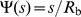 for 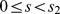 and 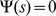 for 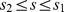 with 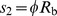. Then 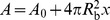 and
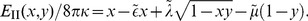
(22)


In both cases I and II, the determinant of the 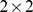 Hessian matrix is always negative within the unit square 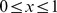, 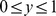, which implies that the local minima of 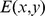 can be found only at the square boundaries. A simple analysis of Eqs. (21) and (22) shows that the local minima of the energy function are located only at the corners of the unit square in the 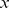
−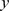 plane. First, 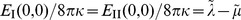. Second, 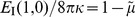, and 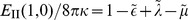. Third, 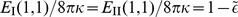. We compare these energies to find the global minimum of the energy function and, in this way, construct the phase diagram in Fig. 2.

Point 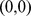 corresponds to the flat membrane state, which is obviously the starting point of the budding process. Point 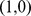 on energy surface 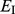 corresponds to an ESCRT-coated bud, whereas point 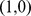 on energy surface 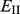 corresponds to coatless buds with radius 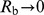. Point 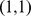 corresponds to a coatless membrane bud with finite radius and ESCRTs localized in the neck. Fluorescence microscopy shows that this state is the end point of the ESCRT-induced budding.

The transition from the initial, flat membrane state at point 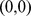 to the end point at 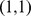 occurs spontaneously with no energy barriers if either (i) 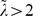 and 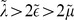, or (ii) 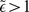 and 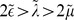. In case (i), the transition occurs on energy surface 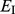. In case (ii), the system evolves on energy surface 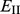. In both cases (i) and (ii) the transitions occur from point 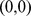 to 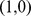 along the 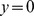 line and next from point 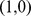 to 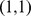 along the 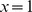 line. We checked that for some parameter combinations, a saddle point can emerge within the unit square, separating local minima at 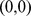 and 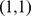. However, if the saddle point is in the unit square, it is higher than 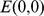. We also checked that for positive parameters 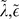 and 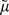, the saddle point cannot merge with point 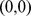. So the barrier-free path should indeed go from point 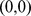 to 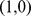 to 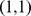.

In the case 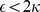, the system stays on energy surface 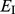. It is then instructive to analyze the energy landscape 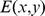 as the circular domain 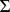 grows. As the domain radius increases beyond 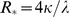, the minimum at 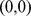 becomes higher than the energy at 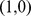. As the domain radius increases to 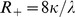, the barrier between 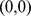 and 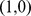 disappears entirely. However, if 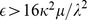, the point 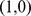 is not a local minimum, with an energy already higher than the minimum at 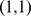. So if budding is induced at the radius 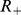, then the ESCRTs either stay on the bud, if 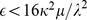, or they coalesce into the neck immediately after bud formation if 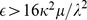.

Another budding scenario is possible if 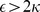 and 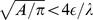. The system evolves then on energy surface 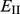 from point 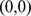 to 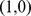 with no energy barriers, which means that flat, ESCRT-coated membrane domains are unstable and small, ESCRT-free buds with radius 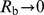 form spontaneously. The mini-buds induced by a strong binding preference 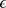 to membrane portions with negative Gaussian curvature are next squeezed bigger by line tension if 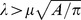. The system evolves then from point 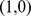 to 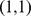. The resulting ESCRT-free buds have a radius 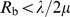. In contrast to the three-stage mechanism, in this budding pathway the ESCRTs never enter the bud lumen.
